# The Urothelial Transcriptomic Response to Interferon Gamma: Implications for Bladder Cancer Prognosis and Immunotherapy

**DOI:** 10.3390/cancers14215295

**Published:** 2022-10-27

**Authors:** Simon C. Baker, Andrew S. Mason, Raphael G. Slip, Pontus Eriksson, Gottfrid Sjödahl, Ludwik K. Trejdosiewicz, Jennifer Southgate

**Affiliations:** 1 Jack Birch Unit of Molecular Carcinogenesis, Department of Biology and York Biomedical Research Institute, University of York, Heslington, York YO10 5DD, UK; 2 Division of Oncology, Department of Clinical Sciences Lund, Lund University, 22184 Lund, Sweden; 3 Department of Translational Medicine, Lund University, 20502 Malmö, Sweden

**Keywords:** bladder cancer, urothelium, interferon gamma, BCG, immunotherapy, immune checkpoint, VISTA, PD-L1

## Abstract

**Simple Summary:**

Bladder cancers are frequently treated by instilling BCG vaccine into the bladder. By provoking inflammation, BCG can lead to the production of a factor called “interferon gamma” and an immune response that can eliminate the cancer. Here, we used normal human uro-epithelial cells that line the urinary tract to establish a specific “signature” of genes activated in response to interferon gamma. When assessed in bladder cancers, the interferon gamma signature was associated with a subset of patients who showed significantly better survival. In normal tissues, immune responses are subject to stop signals called “checkpoints” that prevent runaway inflammation, and this process may prevent immune cells from killing cancer cells. New treatments aimed at tackling immune checkpoints have met with limited success in bladder cancer. In this study, we have identified immune checkpoint genes expressed by normal human uro-epithelial cells that were increased by interferon gamma. This research suggests new targets for use in combination treatments aimed at overcoming immune checkpoints.

**Abstract:**

Interferon gamma (IFNγ) is central to the inflammatory immune response, such as that entrained by BCG immunotherapy for bladder cancer. However, immune-mediated tumour cell killing is subject to modulation by immunoinhibitory “checkpoint” receptors such as PD-L1. We investigated the effects of IFNγ on barrier-forming in vitro-differentiated normal human urothelium using mRNA-sequencing, and showed canonical upregulation of MHC class I/II and de novo expression of the T cell tropic CXCL9-11 chemokines. Normal urothelium constitutively expressed immunoinhibitory B7 family member *VSIR* (VISTA), while *CD274* (PD-L1) expression was induced/upregulated by IFNγ. We generated a urothelial IFNγ response gene signature. When applied to the unsupervised clustering of non-muscle-invasive bladder cancers, the IFNγ-signature predicted longer recurrence-free survival. In muscle-invasive cancers, the IFNγ-signature split the basal/squamous consensus subtype, with significantly worse overall survival when weak or absent. This study offers novel insights into strategies to enhance immunotherapy via the IFNγ and VISTA/PD-L1 nexus.

## 1. Introduction

Instillation of Bacillus Calmette–Guérin (BCG) vaccine into the bladder is widely used for therapy in non-muscle-invasive bladder cancer (NMIBC), where it acts as a potent inducer of inflammation, provoking infiltration by leukocytes, including IFNγ-secreting proinflammatory lymphocytes. These become exposed to tumour neoantigens, eventually resulting in tumour-specific immunity [1]. IFNγ is detectable in urine of patients in the hours after intravesical BCG therapy [2,3,4]. In an orthotopic MB49 mouse bladder cancer model, IFNγ was critical for intrinsic tumour surveillance and an *Ifng*-knockout rendered BCG ineffective [5]. 

Established tumours have presumably evaded immune surveillance mechanisms. As immune evasion may be partial, there should be opportunities for therapeutic strategies aimed at immuno-enhancement. Recent interest has focused on blocking immune inhibitory pathways, with IFNγ again implicated in tumour growth inhibition following immune checkpoint blockade [6]. However, effective immunotherapy strategies need understanding of both the immunoregulatory landscape of the bladder and how expression of immune co-stimulatory and inhibitory factors are modulated by IFNγ. 

In this study, we used our functionally differentiated, mitotically quiescent in vitro model of normal human urothelium [7] to study the innate and IFNγ-induced capacity to modulate immune signalling at the gene expression level. We used the results to generate an IFNγ response signature that we used to study bladder cancer cohorts. This approach is based on the hypothesis that the transcriptomic signature of normal urothelial cells will be applicable to the broadest range of urothelial cancers, irrespective of individual tumour context and profile of genomic damage. The results are informative of the local immune environment in which urothelial cancers develop, and provide new insights for immunotherapy strategies. 

## 2. Materials and Methods

### 2.1. Cell Culture

Six independent Normal Human Urothelial (NHU) cell lines of finite lifespan (non-immortalised) were used. The lines were established as detailed [8], using anonymous discarded tissue from renal transplant surgery, with NHS Research Ethics Committee approval. NHU cell cultures were propagated using Keratinocyte Serum-Free Medium containing bovine pituitary extract, recombinant human EGF and supplemented with 30 ng/mL cholera toxin (KSFMc) [8]. Following expansion, NHU cell cultures were differentiated in the above medium supplemented with 5% adult bovine serum and CaCl_2_ to raise [Ca^2+^] to 2 mM, according to our published methods [9]. Differentiated cultures from all six independent cell lines were incubated with IFNγ (200 U/mL, BioTechne #285-IF) for 7 days, with medium replenished every 48–72 h. Characteristics of urothelial differentiation, including mitotic-quiescence, were largely unaffected by IFNγ, and although there was a significant gain of *KRT6A* expression (log_2_FC = 1.52; q/adj *p* = 0.004), there was no significant loss of transitional epithelial markers and no other indicators of squamous change (Supplementary Table S1).

### 2.2. mRNA Analysis

Total RNA was collected in TRIzol reagent (Invitrogen, Inchinnan UK) and mRNA-sequencing was performed using an Illumina NovaSeq 6000 generating 150 base paired-end reads (Novogene UK, Cambridge, UK; sequencing quality metrics are provided in Supplementary Table S2). All mRNA-sequencing data was deposited at GSE174244. Following standard quality control, gene-level expression values in transcripts per million (TPM) were derived against the Gencode v35 human transcriptome using kallisto v0.46.1 [10]. Differentially expressed genes were identified using the sleuth v0.30.0 [11] implementation of the likelihood ratio test (LRT), accounting for matched genetic backgrounds, to generate Benjamini-Hochberg corrected *q*-values. For volcano plots (performed in R v4.0.4 EnhancedVolcano v1.8.0), fold-change values used a log_2_(TPM + 1) transformation to reduce the influence of low abundance transcripts. This analysis identified 107 genes that were significantly (*q* < 0.05) > 2-fold increased by IFNγ and 48 genes whose expression was significantly (*q* < 0.05) more than halved by IFNγ-treatment of differentiated NHU cell cultures (Supplementary Table S1).

### 2.3. Publicly Available Bladder Cancer Cohort Data

Patient data from four publicly available bladder cancer cohorts were downloaded following instructions in the relevant publications [12,13,14,15]. Two cohorts focussed on NMIBC [12,14] and from these, we extracted the T1 tumours for further analysis. From muscle-invasive bladder cancer (MIBC) cohorts [13,15], tumours classified transcriptomically as “Basal/Squamous” subtype [16] were selected for further analysis based on the high variability we observed in the IFNγ-signature. mRNA-sequencing data was remapped to Gencode v35 human transcriptome using kallisto v0.46.1 where original FASTQ files were available [10]. MIBC gene array data from the Lund cohort was used as deposited at GSE83586 [15] to generate quantile normalised, ComBat adjusted, intensity values merged at the gene level. Expression data from bladder cancer cohorts was visualised using Morpheus (https://software.broadinstitute.org/morpheus, accessed on 23 August 2022).

### 2.4. IFNγ-Signature Generation

The list of genes identified as significantly increased by >2-fold (*q* < 0.05) by IFNγ in differentiated NHU cell cultures was extracted from the TCGA-BLCA [13] and UROMOL2021 [14] cohorts of MIBC and NMIBC, respectively. To refine the signature, Spearman correlation matrices were calculated to compare all genes with one another. Pairwise median Spearman Rho correlations were calculated for each gene independently within each cohort. Genes with an average Spearman Rho value >0.5 across both cohorts (*n* = 33; gene list in Supplementary Table S3) formed a transcriptomic classifier, where all genes were >2-fold increased by IFNγ in vitro and closely correlated in tumours.

Initial evaluation of the IFNγ-signature in tumours was performed on log_2_(TPM + 1) data using hierarchical clustering (based on Euclidean distance and complete linkage). This approach displays the relationship between samples in the full cohorts of NMIBC UROMOL2021 [14] (Supplementary Figure S3) and MIBC TCGA-BLCA [13] (Supplementary Figure S5), respectively.

### 2.5. IFNγ-Signature Analysis

mRNAseq data expressed as TPMs for T1 tumours from the two NMIBC cohorts were combined to create one large cohort for analysis. For the MIBC cohorts, to allow integration of data acquired by different methods (TCGA-BLCA mRNA-sequencing [13] with Lund gene arrays [15]) each cohort was clustered separately into IFNγ-signature high and low groups. Classification of tumours was performed on log_2_(TPM + 1) data using Euclidean distance k means clustering into two groups of T1 NMIBC tumours (from the UROMOL2021 [14] and Northwestern Memorial Hospital [12] cohorts) and Basal/Squamous classified [16] MIBC tumours (TCGA-BLCA [13]). Classification of Basal/Squamous MIBC tumours from the Lund [15] cohort was performed using Euclidean distance k means clustering into two groups on log-transformed ComBat adjusted, gene-level intensity values.

A single-value IFNγ-signature score was derived by unit-length scaling the log_2_(TPM + 1) data (or log-transformed expression values for the Lund cohort [15]) for each gene. Unit-length scaled data for the genes in the signature were then summed on a per patient basis before being re-scaled from 0–1. This derived a single value IFNγ-signature score that could be ranked to generate Spearman Rho values in comparison with ranked gene expression values in the various cohorts. The single-value IFNγ-signature scores for the combined T1, TCGA-BLCA and Lund cohorts are in Supplementary Tables S4, S6 and S7, respectively.

Survival analysis was performed in Prism v9.3.1 (Graphpad). T1 NMIBC tumours and Basal/Squamous classified MIBC tumours from the different cohorts were pooled to support Kaplan–Meier NMIBC Recurrence Free Survival (RFS) and MIBC Overall Survival (OS) analysis, respectively. Statistical significance of the difference between Kaplan–Meier curves for IFNγ-signature high and low groups was performed using Mantel-Cox and Gehan-Breslow-Wilcoxon tests, with hazard ratios calculated using both Mantel-Haenszel and log rank approaches. A Cox proportional hazards regression analysis was performed using the pseudo-continuous unit-length scaled IFNγ-signature scores to demonstrate that the effects of IFNγ-signalling persisted without dichotomising the tumours.

## 3. Results

### 3.1. The Urothelial IFNγ Response 

**Figure 1:**
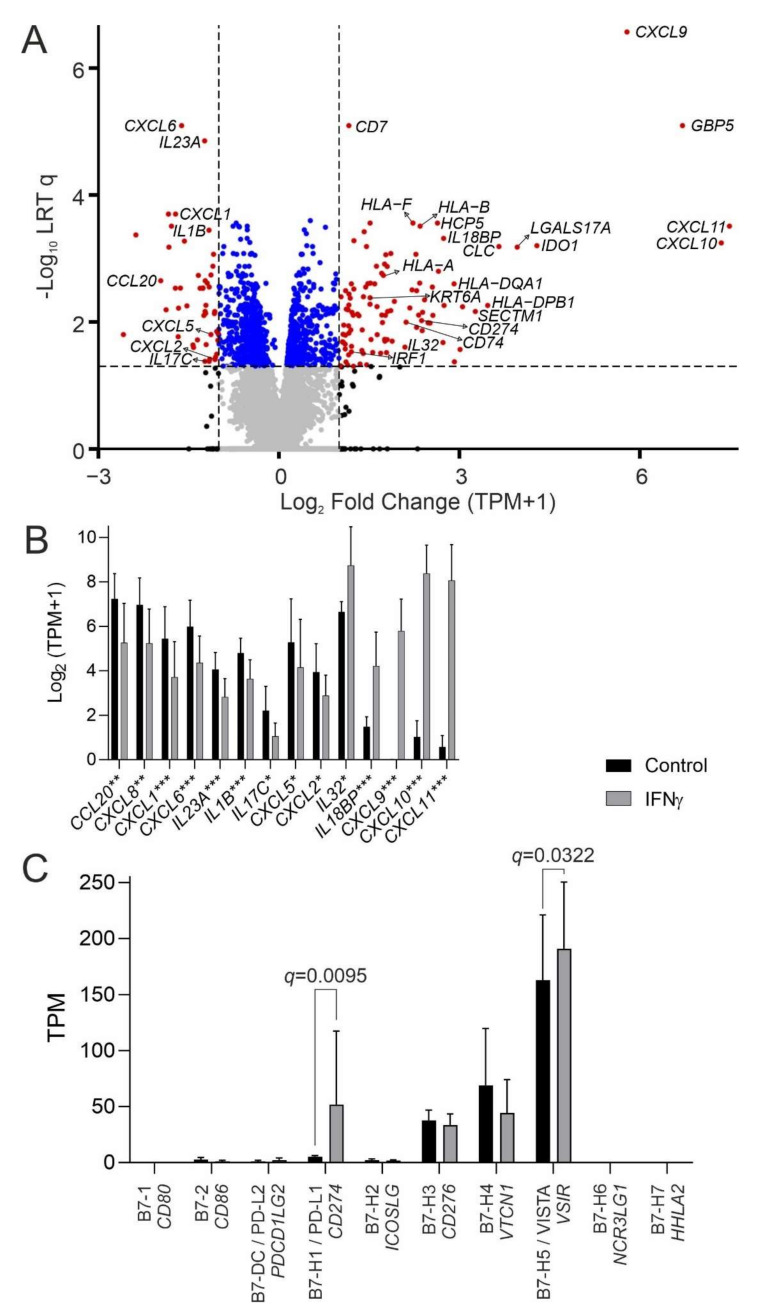
Response of differentiated normal human urothelial cell cultures to 200 U/mL IFNγ for seven days. Panels show data from six independent donors. (**A**) mRNA-sequencing volcano plot for the IFNγ-induced normal urothelial transcriptome. Data are expressed as transcripts per million (TPM). Significance was assessed using a likelihood ratio test (LRT) to generate Benjamini-Hochberg corrected *q*-values. (**B**) Shift in cytokine and chemokine gene expression induced by IFNγ. Stars indicate >2-fold changes with significance * = *q* < 0.05, ** = *q* < 0.01 and *** = *q* < 0.001. (**C**) Urothelial expression of the B7 family of immunoregulatory accessory protein genes. The immune co-stimulatory molecule genes *CD80* and *CD86* were not expressed. *CD274* (PD-L1) was significantly induced by IFNγ (mean fold change = 5.2; *q* = 0.0095). *VSIR* (VISTA) was abundantly expressed and significantly induced by IFNγ (mean fold change = 1.2; *q* = 0.0322). HGNC gene names are italicized, common protein names are shown in normal font.

The IFNγ receptor genes (*IFNGR1* and *IFNGR2*) were abundantly expressed by urothelium (Supplementary Figure S1). mRNA-sequencing of mitotically quiescent (G_0_-arrested), in vitro-differentiated normal human urothelium from six independent donors incubated with 200 U/mL IFNγ for seven-days identified 107 genes significantly (*q* < 0.05) >2-fold increased (Figure 1A and Supplementary Table S1).

IFNγ induced a shift in expression of chemokine and cytokine genes implicated in immune recruitment, with loss (*CCL20*, *CXCL8*, *CXCL1*, *CXCL6*, *IL23A*, *IL1B*, *IL17C*, *CXCL5*, *CXCL2*) and gain (*CXCL11*, *CXCL10*, *CXCL9*, *IL18BP*, *IL32*) of different signalling factors (Figure 1B). 

In all six donors, irrespective of haplotype, IFNγ induced gain of human leukocyte antigen (HLA) gene expression associated with major histocompatibility complex (MHC) class I and class II, including the associated components β2-microglobulin (*B2M*), MHC class II invariant chain (*CD74*), trans-activator (*CIITA*) and the antigen peptide transporters *TAP1* and *TAP2* (Supplementary Figure S2). 

Immune co-stimulatory B7 family *CD80* and *CD86* genes were absent and were not inducible by IFNγ. *CD274* (PD-L1) was expressed minimally, but strongly induced by IFNγ (5.2-fold). By contrast, the inhibitory B7 family member *VSIR* (VISTA) was highly expressed constitutively and further upregulated by IFNγ (Figure 1C). 

### 3.2. Application of the IFNγ Response Signature to Tumours

We applied a curated urothelial in vitro IFNγ-response gene set (Supplementary Table S3) to the unsupervised clustering of bladder cancer cohorts to derive an “IFNγ-signature” score.

**Figure 2:**
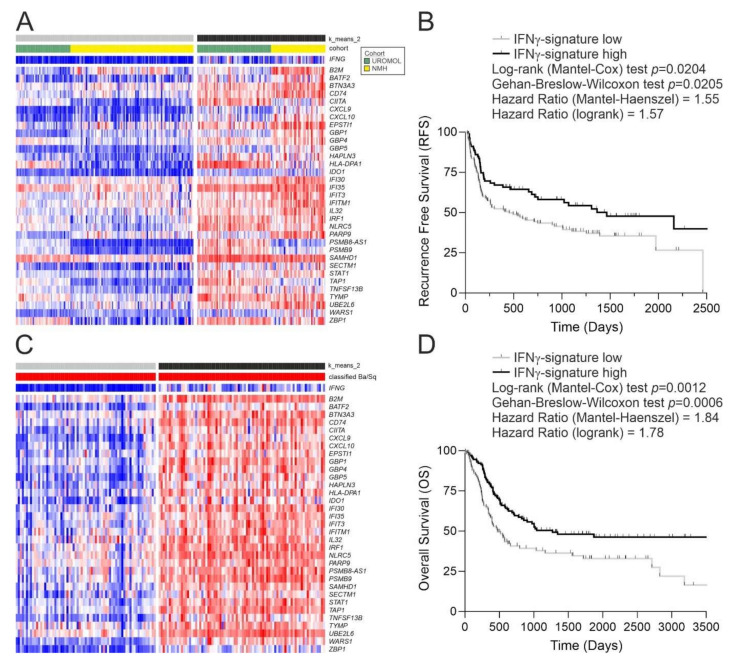
IFNγ signature expression in bladder cancer. (**A**) Heatmap and k means clustering based on expression of the IFNγ-signature genes in the T1 tumours of UROMOL2021 and Northwestern Memorial Hospital (NMH) cohorts (*n* = 236). The IFNγ-signature shows a Spearman rank correlation of 0.66 (*p* = 4 × 10^−31^) with the *IFNG* gene in T1 tumours. The full UROMOL2021 cohort is in Supplementary Figure S3 [14]. The separate NMH T1 cohort (*n* = 99; [12]) is shown as Supplementary Figure S4. (**B**) Kaplan–Meier analysis of IFNγ-signature high and low T1 tumours with survival data from UROMOL2021 and NMH cohorts combined (*n* = 201). There were no events recorded beyond the Kaplan–Meier curve truncation. A Cox proportional hazards regression was also performed using the unit length-scaled IFNγ-signature values from T1 tumours, which predicted a recurrence hazard ratio of 3.174 *p* = 0.0230 (Supplementary Table S5). (**C**) Heatmap for expression of the IFNγ-signature in the *n* = 151 Basal/Squamous group of TCGA MIBC tumours classified (red) according to the consensus report [16], with the full cohort shown in Supplementary Figure S5 [13]. The IFNγ-signature shows a Spearman rank correlation of 0.87 (*p* = 3.85 × 10^−47^) with the IFNG gene in Ba/Sq TCGA-BLCA tumours. Similar analysis for the Lund MIBC cohort (*n* = 88; [15]) is shown in Supplementary Figure S6. (**D**) Kaplan–Meier analysis of the IFNγ-signature in Basal/Squamous tumours from TCGA and Lund MIBC cohorts combined (*n* = 232). There were no events recorded beyond where the curve is truncated. A Cox proportional hazards regression performed using the unit length-scaled IFNγ-signature values from both cohorts predicted a hazard ratio of 4.846 *p* = 0.0006 (Supplementary Table S8).

In NMIBC, T1 tumours (combined from Northwestern Memorial Hospital (NMH) [12] and UROMOL2021 [14]) were split by the IFNγ-signature into high and low groups (Figure 2A), with tumour recurrence being 50% more likely in patients in the IFNγ-signature^low^ group (Figure 2B). A Cox proportional hazards regression analysis of the pseudo-continuous unit length-scaled IFNγ-signature scores in T1 tumours predicted a recurrence hazard ratio of 3.174 *p* = 0.0230 (Supplementary Tables S4 and S5). 

Analysis of TCGA-BLCA data [13] revealed that MIBC lacking the IFNγ-signature were most likely to be graded histologically as lymphocyte-negative and of “luminal papillary” molecular sub-type according to the consensus classification [16] (Supplementary Figure S5). By contrast, MIBC molecularly classified as “basal/squamous” split into two clear subgroups (Figure 2C). To examine any difference in outcomes associated with the IFNγ-signature in basal/squamous tumours, the Lund MIBC cohort [15] was analysed in parallel (Supplementary Figure S6). Overall survival for basal/squamous tumours (pooled from TCGA-BLCA and Lund cohorts) was significantly lower in tumours with a weak IFNγ-signature (Figure 2D). A high survival hazard ratio of 4.846 *p* = 0.0006 was predicted by Cox proportional hazards regression based on the pseudo-continuous IFNγ-signature scores from both cohorts (Supplementary Tables S6–S8)

### 3.3. Bladder Cancer Neoantigens

**Figure 3:**
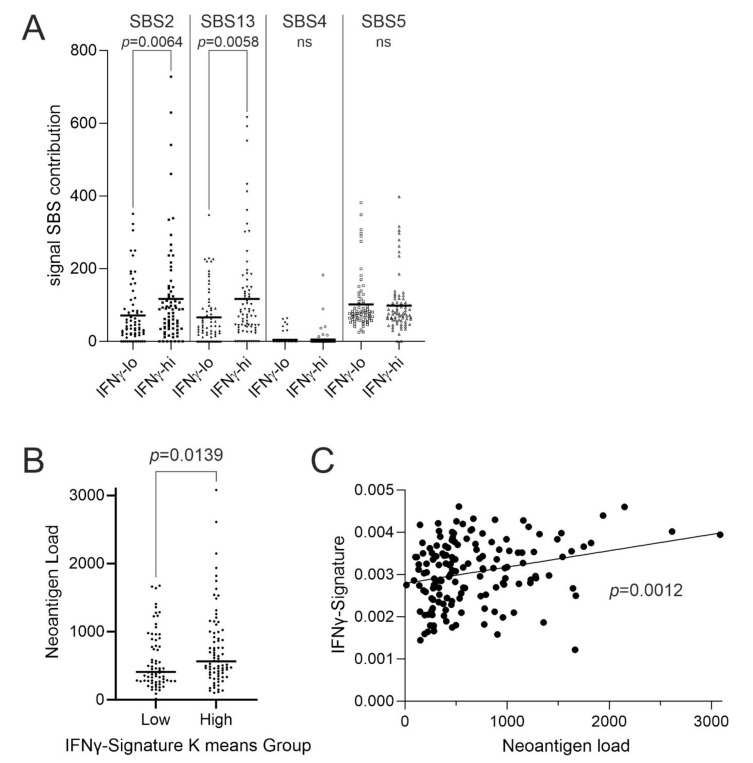
Analysis of mutations using paired exome-sequencing data from TCGA-BLCA. There was no significant over-mutation of individual genes in association with the IFNγ-signature. (**A**) Assessment of mutational processes revealed specific evidence of APOBEC-driven changes (Catalogue of Somatic Mutations in Cancer “COSMIC” single base substitution signatures “SBS2” and “SBS13”) in IFNγ^high^ tumours, where lack of enrichment of single base substitutions from other causes (COSMIC SBS4 and SBS5), indicated no overall increase in mutational rates. Significance was analysed by Mann–Whitney U-test. (**B**) Analysis of Basal/Squamous tumours of TCGA-BLCA cohort showed a significantly higher predicted neoantigen load in the context of higher IFNγ-signalling (*p* = 0.0139). (**C**) Linear regression analysis confirmed a significant relationship between predicted neoantigen load and IFNγ-signature score (*p* = 0.0012).

Based on the observation of variance in the IFNγ-signalling of basal/squamous MIBC tumours, we hypothesised that this was related to the strength of the immune response provoked by the diverse neoantigen loads of these tumours. The APOBEC3A and APOBEC3B cytosine deaminases are the main mutators of bladder cancer genomes (evidenced as COSMIC SBS2 and SBS13 mutational signatures in 93% of bladder tumours [13]) and therefore account for most of the neoantigen load in bladder tumours. IFNγ-signature^high^ basal/squamous MIBC tumours had significantly enriched genomic damage from APOBEC enzymes (Figure 3A). Widespread APOBEC damage leads to tumour suppressor loss and creates neoantigens, which were significantly more abundant in IFNγ-signature^high^ basal/squamous tumours (Figure 3B). There was a significant correlation between neoantigen load (13) and IFNγ signature expression (Figure 3C). 

## 4. Discussion

This study used a curated gene set representing the in vitro response of human urothelium to IFNγ to provide new understanding of signalling in bladder tumours. A lack of this urothelium-specific IFNγ-signature was associated with increased risk of progression in T1 tumours and decreased overall survival in the basal/squamous sub-type of MIBC.

Urothelium functions as a physical urinary barrier and plays a primary role in defence against uropathogens through innate Toll-like receptor mechanisms [17]. The signalling involved in excluding or recruiting immunocytes into the urothelium is poorly understood, but is important for understanding how the urothelium interacts with the innate and adaptive immune systems for functions such as tumour surveillance. The latter is particularly relevant given the potential for immunotherapies to overcome host anergy to tumours. Our finding that IFNγ induced urothelial expression of the *CXCL9*, *CXCL10* and *CXCL11* chemokine genes is pertinent, as the CXCL9/10/11-CXCR3 axis is a critical regulator of leukocyte migration, differentiation and activation, including recruitment of effector T cells [18]. We have shown previously that *CXCL10/11* are highly induced following exposure of normal urothelium to BK polyomavirus, where they were the only genes to be further upregulated, rather than repressed, by exogenous IFNγ [19]. This demonstrates a virally initiated positive feedback to the recruitment of further IFNγ-producing cells and suggests that attenuated BK virus could be used to generate a pro-inflammatory, IFNγ-rich local environment akin to that induced by BCG.

Gains in transcript expression of β2-microglobulin (*B2M*), MHC class II invariant chain (CD74) and trans-activator (*CIITA*), as well as the antigen peptide transporters *TAP1* and *TAP2*, imply an induced role in modulating antigen-presentation to effector T cells. However, urothelial cells are unlikely to act as professional antigen-presenting cells (ie cells capable of priming naive T cells), because the co-stimulatory B7 family CD80 and CD86 genes were not expressed, and were not inducible by IFNγ. Thus, it seems likely that T cell priming, leading to the development of cytotoxic responses, occurs following BCG-induced recruitment of accessory cells or in the draining lymph nodes, whereas activation of primed lymphocytes can occur within the bladder microenvironment without the requirement for accessory cells. 

Inhibitory members of the B7 family and the PD-L1 (CD274) immune checkpoint act to counter runaway immunocyte activation through inhibition of T cell activation and promotion of regulatory CD4 (Treg) cell activity. Indeed, blocking antibodies against PD-L1 have been trialled clinically to disinhibit tumour-specific cytotoxic lymphocytes, but with limited success [20]. Paradoxically, we found that *CD274* (PD-L1) was strongly induced by IFNγ. In addition, the inhibitory B7 family member *VSIR* (VISTA) was expressed constitutively by normal human urothelium, and further upregulated by IFNγ. This holds potential significance for immunotherapy as VISTA expression in the bladder has received very little attention to date [21]. Our finding suggests that normal urothelium, and likely its malignant counterpart, presents a locally immunosuppressive environment, with induction of PD-L1 by IFNγ acting as a negative feedback loop.

Cancer subtyping is usually performed by unsupervised clustering of the most statistically informative genes detected as transcripts in extracted tumour tissue, the latter representing a heterogeneous mix of tumour, stromal and immune cells. Although the *IFNG* transcript itself can be detected in cancer cohorts, it shows weak sensitivity and, being a diffusible factor, IFNγ can elicit a response in the tumour even when originating from peritumoural leukocytes. This led us to investigate whether a curated urothelial in vitro IFNγ-response gene set applied to the unsupervised clustering of bladder cancer cohorts to yield an “IFNγ-signature” score would be more informative of tumour IFNγ response. MIBC classified as luminal papillary subtype generally lacked the IFNγ-signature and were devoid of lymphocyte infiltration. By contrast, there was a clear split of the basal/squamous tumours into high and low IFNγ-signature groups, which correlated with markedly different survival profiles. Our finding suggests for the first time that the basal/squamous subtype of MIBC should be regarded as two biologically distinct entities.

Genomically, a characteristic of the IFNγ-signature^high^ basal/squamous subgroup is that the cancers carry elevated signatures of Apolipoprotein B mRNA Editing Catalytic Polypeptide-like (APOBEC) mediated mutagenesis [22]. APOBEC-activity is associated with a high mutation load resulting in the acquisition of neoantigens [23]. In an inflammatory context, neoantigens would be expected to induce antigen-specific T cell immunity and IFNγ-signalling. However, our data suggest that normal and malignant urothelium are likely to suppress T cell activation through PD-L1/VISTA checkpoint inhibition, thereby inhibiting type-1 anti-tumour immune responses. This implies that a balance becomes established between activated tumoricidal effector cells and regulatory/immunosuppressive elements; the therapeutic challenge is to swing the balance in favour of tumour cytotoxicity. 

In conclusion, this study suggests a reconsideration of immunotherapy protocols for bladder cancers based on IFNγ-response signature. Whilst beyond the scope of this present study, the verification of candidate markers on tumours from patients that did or did not respond to BCG will be important for validating the clinical utility of our approach. The IFNγ-signature^high^ basal/squamous MIBC tumours identified here appear to represent a target for immune checkpoint blockade. However, expression of PD-L1 is likely to be too low to provide an effective target in IFNγ-signature^low^ tumours and VISTA would be a more appropriate target. For NMIBC, our results suggest that BCG therapy would be most effective when combined with blocking of both the innate (VISTA) and inducible (PD-L1) immune checkpoints to take maximum advantage of the effects of BCG-induced IFNγ secretion. 

## Data Availability

Patient data from four publicly available bladder cancer cohorts were downloaded following instructions in the relevant publications [12,13,14,15]. All mRNA-sequencing data produced as part of this study is deposited at GSE174244 (https://www.ncbi.nlm.nih.gov/geo/query/acc.cgi?acc=GSE174244, accessed on 23 August 2022) and all other study relevant data are contained within the article or supplementary material.

## Supplementary Materials

The following supporting information can be downloaded at: https://www.mdpi.com/article/10.3390/cancers14215295/s1. Figure S1: Urothelial expression of both IFNγ receptor isoform genes was high but significantly reduced by IFNγ treatment; Figure S2: Gain in expression of a broad range of human leukocyte antigen (HLA) genes; Figure S3: The full UROMOL2021 NMIBC cohort (*n* = 535; [14]) expression of the IFNγ-signature genes; Figure S4: Heatmap and k means clustering based on expression of the IFNγ-signature in the T1 tumours of the Northwestern Memorial Hospital (NMH) cohort (*n* = 99; shown with the classifier from the original report [12]); Figure S5: (A) The full TCGA-BLCA MIBC cohort (*n* = 404; [13]) expression of the IFNγ-signature genes. Tumours were split using hierarchical clustering based on Euclidean distance with complete linkage. The *IFNG* gene itself is not part of the signature and has a limited sensitivity for detecting IFNγ signalling; however, it does show significant correlation with the IFNγ-signature (Spearman Rho = 0.83; p = 2.86 × 10^−102^). Tumours are coloured according to the 2019 consensus classification into six subtypes (Basal/Squamous, Luminal Non-specified, Luminal Papillary, Luminal Unstable, Neuroendocrine-like and Stroma-rich) [16]. The heatmap shows diversity in the IFNγ-signature within the Basal/Squamous group of MIBC and so these tumours were evaluated further (Figure 2C). (B) Histological grading of lymphocyte invasion of TCGA-BLCA tumours, showing significant (Chi square = 56.63; df = 4; p = 1.48 × 10^−11^) differences, including that IFNγ-signature low tumours were more likely to contain no lymphocytes; Figure S6: Basal/Squamous classified tumours from the Lund MIBC cohort (*n* = 88) showing expression of the IFNγ-signature genes (where present on the gene arrays employed); Table S1: Significantly (Benjamini-Hochberg corrected *q*-value < 0.05) >2 -fold changing gene list; Table S2: RNA sequencing (RNA-seq) quality metrics for samples mapped against the Gencode v35 human transcriptome using kallisto v0.46.1; Table S3: IFNg Signature Gene List; Table S4: NMIBC T1 Cohort (combined UROMOL and NMH mRNAseq) data including patient identifiers and corresponding IFNg signature scores; Table S5: T1 NMIBC Cox proportional hazards regression based on IFNg signature scores; Table S6: The Cancer Genome Atlas (TCGA) MIBC Cohort patient identifiers with corresponding IFNg signature scores (Kamoun et al. 2019 classifications are also provided); Table S7: Lund MIBC Cohort patient identifiers with corresponding IFNg signature scores (Kamoun et al. 2019 classifications are also provided); Table S8: MIBC Cox proportional hazards regression based on IFNg signature scores.
