# Supplementary material for: The Urothelial Transcriptomic Response to Interferon Gamma: Implications for Bladder Cancer Prognosis and Immunotherapy
Source: Cancers (Basel). 2022 Oct 27;14(21):5295. doi: 10.3390/cancers14215295 (PMC9654607; doi:10.3390/cancers14215295)
Supplement: Supplementary file 1 [file cancers-14-05295-s001.zip › cancers-1907202-supplementary/Supplementary Information.pdf]

Supplementary Figures

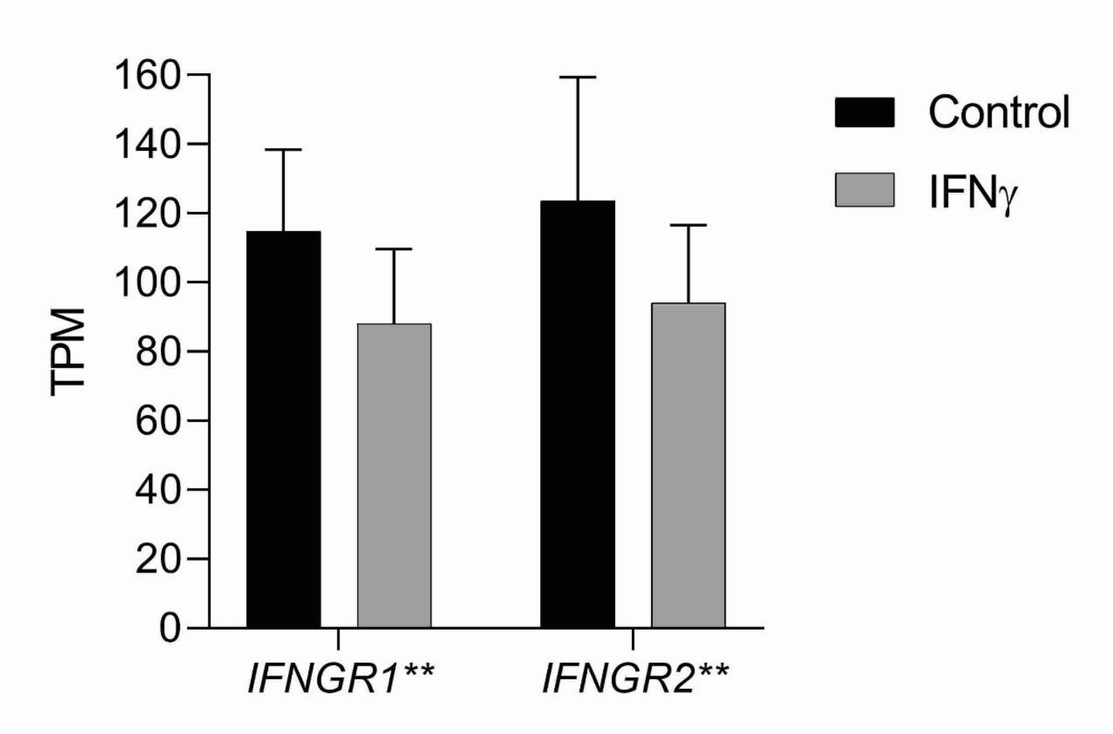

*Figure S1 – Urothelial expression of both IFNγ receptor isoform genes was high but significantly reduced by IFNγ treatment. Mean log_2 fold changes were -0.39 and -0.38 for IFNGR1 and IFNGR2, respectively. Stars following gene names indicate significance **=p<0.01.*

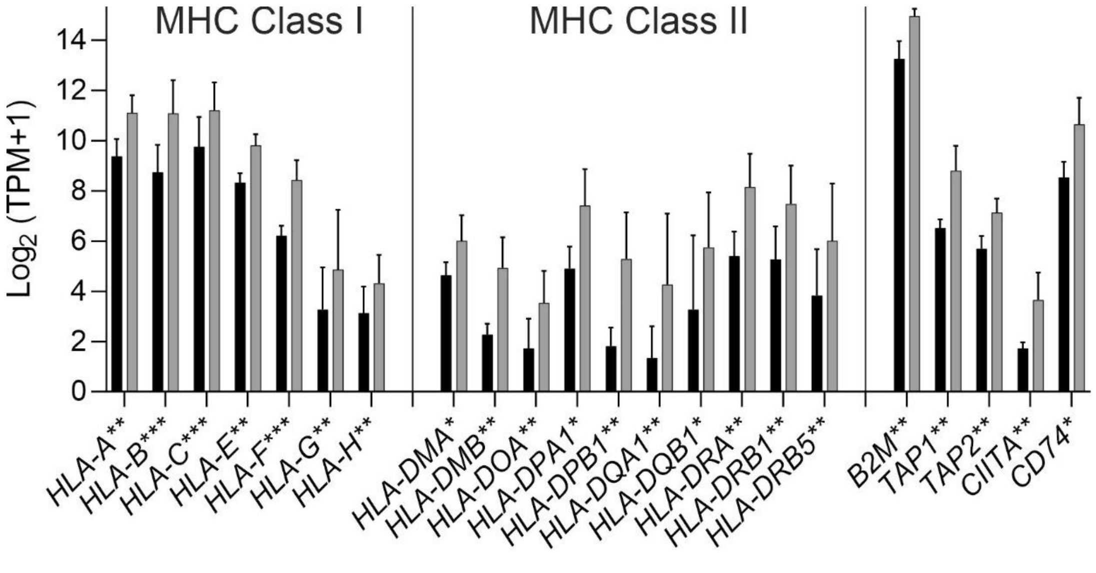

*Figure S2 – Gain in expression of a broad range of human leukocyte antigen (HLA) genes. This study used urothelial cells derived from six independent donors. Stars following gene names indicate >2-fold changes with significance *=q<0.05, **=q<0.01 and ***=q<0.001.*

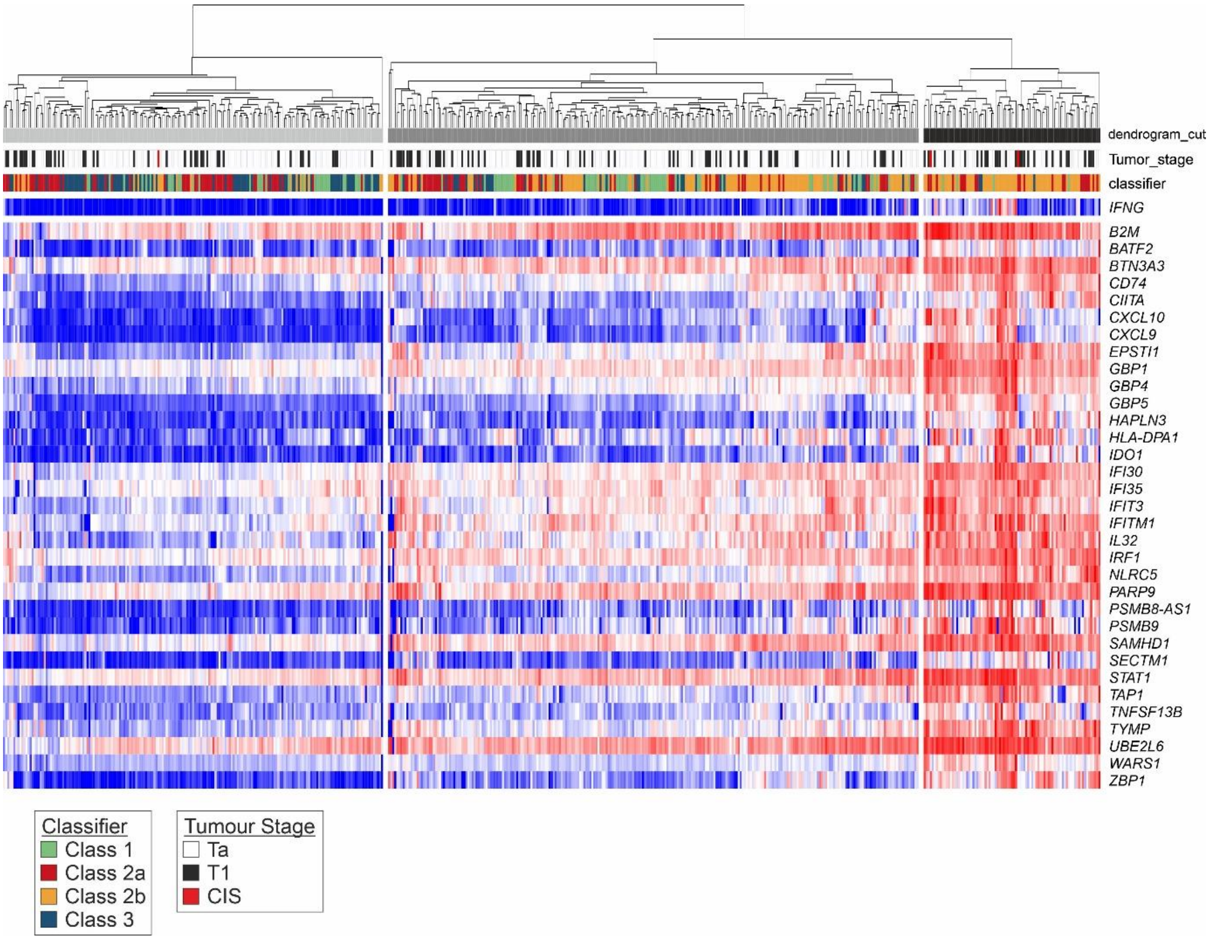

Figure S3 – The full UROMOL2021 NMIBC cohort (n=535; [1]) expression of the IFNγ-signature genes. Tumours were split using hierarchical clustering based on Euclidean distance with complete linkage. The IFNG gene is not part of the signature but is included above to show the lack of sensitivity when relying on IFNG transcript abundance alone. IFNG transcript abundance was significantly correlated with the IFNγ-signature (Spearman Rho=0.57; p=1.02x10^{-46}). Tumours are coloured according to the 2021 classification into four subtypes (Class 1, Class 2a, Class 2b and Class 3) [1]. The heatmap shows enrichment of IFNγ responsive gene expression in a subset of the Class 2b group of NMIBC. Lindskrog et al. identified Class 2b as being the most immune-infiltrated tumours [1]. Class 2a tumours were predominantly IFNγ-signature^{low} and had the highest recurrence rate in the original publication [1].

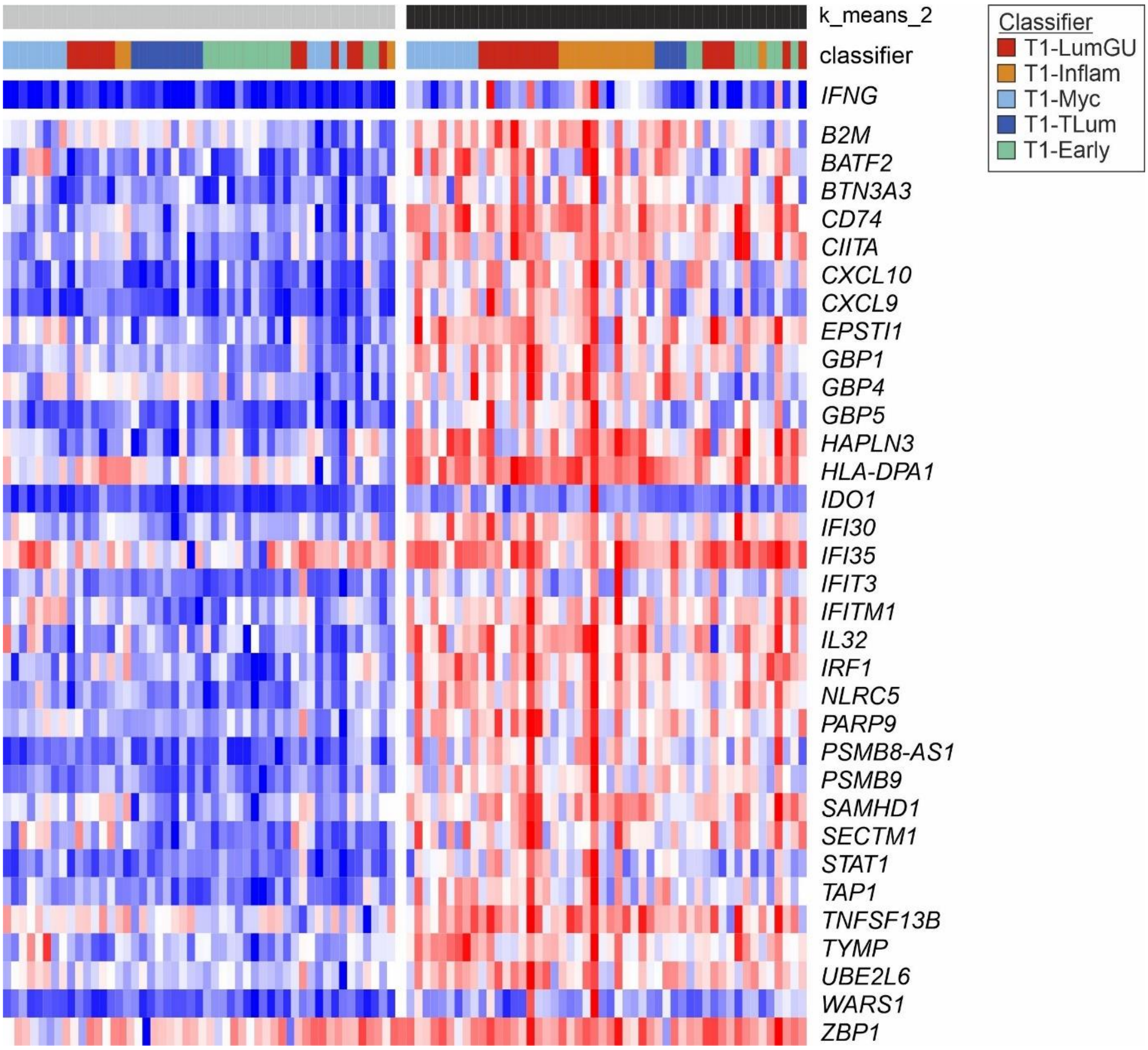

Figure S4 – Heatmap and k means clustering based on expression of the IFNγ-signature in the T1 tumours of the Northwestern Memorial Hospital (NMH) cohort (n=99; shown with the classifier from the original report [2]). The IFNγ-signature shows a Spearman rank correlation of 0.67 ($p=1.66 \times 10^{-14}$) with the IFNG gene in this cohort.

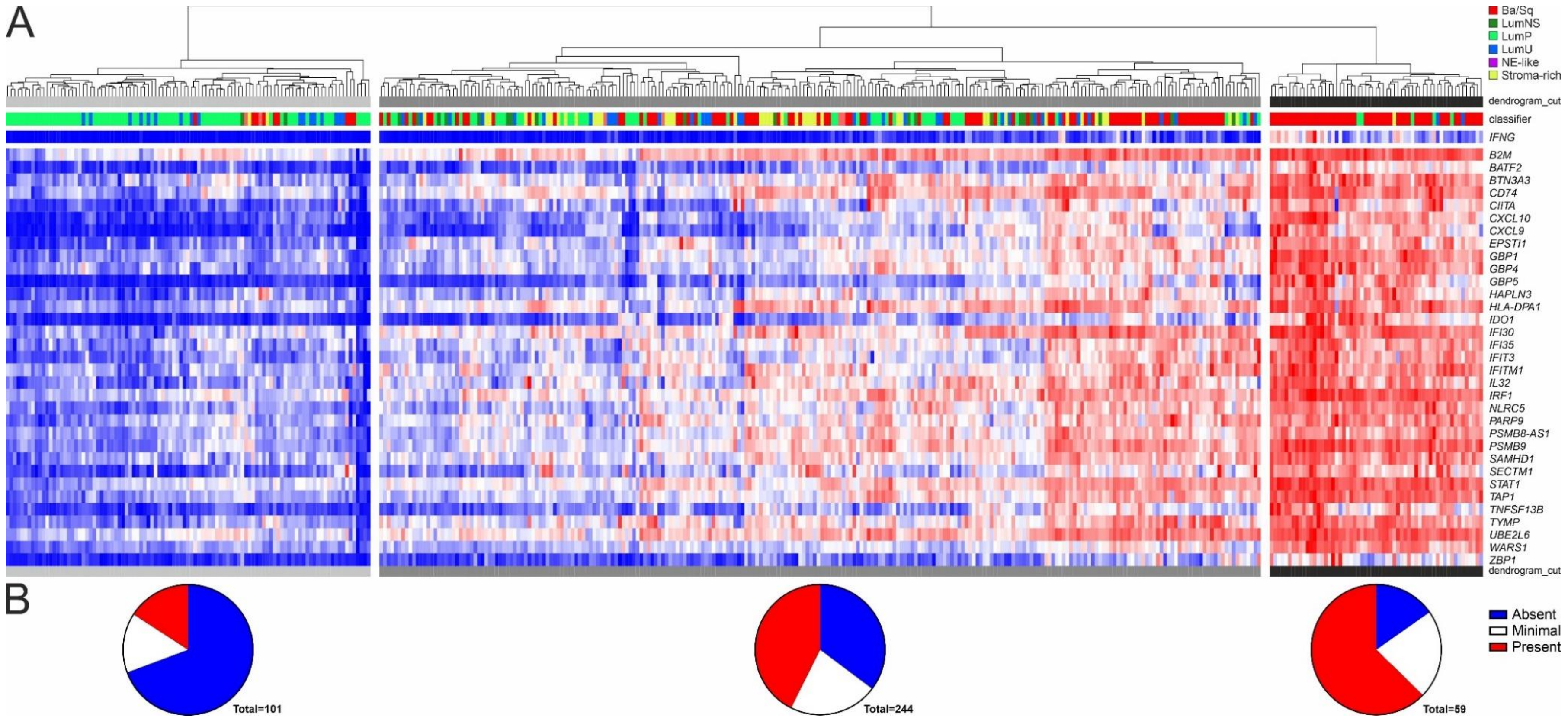

Figure S5 – (A) The full TCGA-BLCA MIBC cohort (n=404; [3]) expression of the IFNγ-signature genes. Tumours were split using hierarchical clustering based on Euclidean distance with complete linkage. The IFNG gene itself is not part of the signature and has a limited sensitivity for detecting IFNγ signalling; however, it does show significant correlation with the IFNγ-signature (Spearman Rho=0.83; $p=2.86x10^{-102}$). Tumours are coloured according to the 2019 consensus classification into six subtypes (Basal/Squamous, Luminal Non-specified, Luminal Papillary, Luminal Unstable, Neuroendocrine-like and Stroma-rich) [4]. The heatmap shows diversity in the IFNγ-signature within the Basal/Squamous group of MIBC and so these tumours were evaluated further (Figure 2C). (B) Histological grading of lymphocyte invasion of TCGA-BLCA tumours, showing significant (Chi square=56.63; df=4; $p=1.48x10^{-11}$) differences, including that IFNγ-signature low tumours were more likely to contain no lymphocytes.

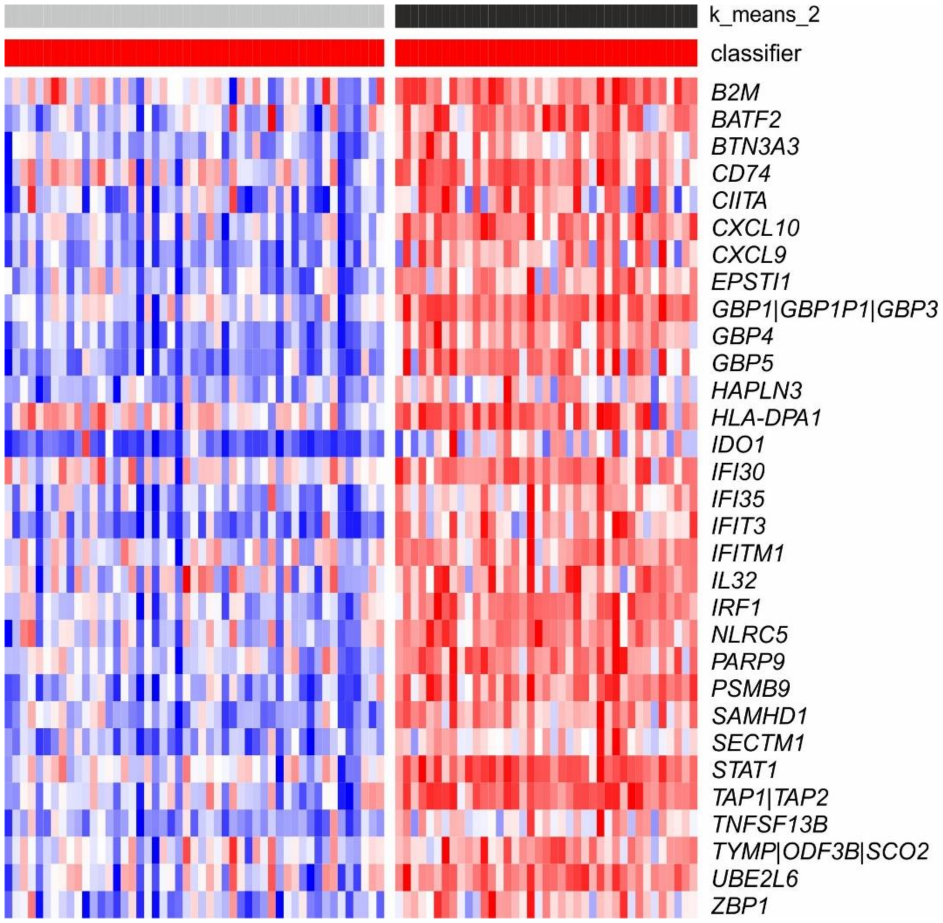

Figure S6 – Basal/Squamous classified tumours from the Lund MIBC cohort (n=88) showing expression of the IFNγ-signature genes (where present on the gene arrays employed). Multiple gene names indicate the gene array probes bind multiple targets. There was no specific probe for the IFNG gene and so correlation with the signature could not be calculated in this cohort.

Supplementary References

1. Lindskrog, S.V.; Prip, F.; Lamy, P.; Taber, A.; Groeneveld, C.S.; Birkenkamp-Demtroder, K.; Jensen, J.B.; Strandgaard, T.; Nordentoft, I.; Christensen, E., et al. An integrated multi-omics analysis identifies prognostic molecular subtypes of non-muscle-invasive bladder cancer. *Nat Commun* **2021**, *12*, 2301, doi:10.1038/s41467-021-22465-w.
2. Robertson, A.G.; Groeneveld, C.S.; Jordan, B.; Lin, X.; McLaughlin, K.A.; Das, A.; Fall, L.A.; Fantini, D.; Taxter, T.J.; Mogil, L.S., et al. Identification of Differential Tumor Subtypes of T1 Bladder Cancer. *Eur Urol* **2020**, *78*, 533-537, doi:10.1016/j.eururo.2020.06.048.
3. Robertson, A.G.; Kim, J.; Al-Ahmadie, H.; Bellmunt, J.; Guo, G.; Cherniack, A.D.; Hinoue, T.; Laird, P.W.; Hoadley, K.A.; Akbani, R., et al. Comprehensive Molecular Characterization of Muscle-Invasive Bladder Cancer. *Cell* **2017**, *171*, 540-556 e525, doi:10.1016/j.cell.2017.09.007.
4. Kamoun, A.; Reynies, A.; Allory, Y.; Sjodahl, G.; Gordon Robertson, A.; Seiler, R.; Hoadley, K.A.; Groeneveld, C.S.; Al-Ahmadie, H.; Choi, W., et al. A Consensus Molecular Classification of Muscle-invasive Bladder Cancer. *Eur Urol* **2019**, 10.1016/j.eururo.2019.09.006, doi:10.1016/j.eururo.2019.09.006.
